# Absorbable hemostatic hydrogels comprising composites of sacrificial templates and honeycomb-like nanofibrous mats of chitosan

**DOI:** 10.1038/s41467-019-10290-1

**Published:** 2019-05-24

**Authors:** Eric E. Leonhardt, Nari Kang, Mostafa A. Hamad, Karen L. Wooley, Mahmoud Elsabahy

**Affiliations:** 1 Departments of Chemistry, Chemical Engineering, Materials Science & Engineering, and The Laboratory for Synthetic-Biologic Interactions, Texas A&M University, College Station, TX 77842-3012 USA; 2 Department of Surgery, Faculty of Medicine, Assiut University, Assiut, 71515 Egypt; 3 Department of Pharmaceutics and Assiut International Center of Nanomedicine, Al-Rajhy Liver Hospital, Assiut University, Assiut, 71515 Egypt; 4 Misr University for Science and Technology, 6th of October City, 12566 Egypt

**Keywords:** Translational research, Polymer chemistry, Nanocomposites, Biomaterials, Nanoscience and technology

## Abstract

The development of hemostatic technologies that suit a diverse range of emergency scenarios is a critical initiative, and there is an increasing interest in the development of absorbable dressings that can be left in the injury site and degrade to reduce the duration of interventional procedures. In the current study, β-cyclodextrin polyester (CDPE) hydrogels serve as sacrificial macroporous carriers, capable of degradation under physiological conditions. The CDPE template enables the assembly of imprinted chitosan honeycomb-like monolithic mats, containing highly entangled nanofibers with diameters of 9.2 ± 3.7 nm, thereby achieving an increase in the surface area of chitosan to improve hemostatic efficiency. In vivo, chitosan-loaded cyclodextrin (CDPE-Cs) hydrogels yield significantly lower amounts of blood loss and shorter times to hemostasis compared with commercially available absorbable hemostatic dressings, and are highly biocompatible. The designed hydrogels demonstrate promising hemostatic efficiency, as a physiologically-benign approach to mitigating blood loss in tissue-injury scenarios.

## Abstract (web summary)

Sacrificial templates are used for a range of different applications. Here, the authors synthesise a hyperbranched cyclodextrin polyester template with large void spaces, which is degraded in physiological conditions, and use it to create high surface area chitosan scaffolds for haemostatic applications.

## Introduction

Hemorrhage is a leading cause of death in traumatic injuries^1^, the fourth-leading cause of death in the United States with a total cost of 671 billion USD in 2013^2^. Mitigating blood loss is, therefore, a vital initiative, serving as the impetus for the development of diverse hemostatic technologies and biomaterials for emergency scenarios, not only in both civilian and military traumatic settings, but also during variable therapeutic interventions^3^. Hemostatic agents derived from chitosan, a naturally-derived polysaccharide produced from the deacetylation of chitin, have been widely studied for their fast-acting and localized hemostatic effects^4^. Chitosan is also well known for its antimicrobial properties, and its protection against a wide range of bacteria enhances its appeal as a bandage, where it has found use as the active component in a number of commercial wound dressings, including HemCon®, Chitodine®, TraumaDEX®, Celox™, and others^4–6^. The efficacy of chitosan-based hemostatic agents has been established in significantly improving survival rates and the reduction of blood loss, by accelerating and strengthening blood clots^7,8^. As these technologies rely on a high surface area to promote interaction with platelets and coagulation factors, maximizing the surface area of these chitosan-based materials is a critical initiative for improving their effectiveness^9^. Reported methods to increase the surface area of chitosan-based biomaterials include micellization^10^ and incorporation into porous templates^11,12^. The incorporation of chitosan into a template, serving as a delivery vessel, is an especially attractive option, as it simplifies administration. However, products that rely on the loading of chitosan into non-degradable carriers (e.g., gauze) necessitate further procedures to remove the bandage and may lead to potential complications^13^, thus rationalizing the development of physiologically-benign, absorbable chitosan delivery agents.

Absorbable hemostatic dressings, such as Surgicel®, Curaspon®, and others, are generally composed of gelatin, oxidized regenerated cellulose, collagen, etc^14–16^. Although, in principle, chitosan is a degradable material, chitosan hemostatic powder is not considered bioabsorbable, as it forms large aggregates, generally requires acidic conditions for dissolution, and, therefore, must be removed from the wound before surgical repair^17^. Nanoscale assembly of chitosan may aid in enhancement of the hemostatic efficiency and prevention of aggregate formation in physiological milieu; although, such assembly by the loading of chitosan into bioabsorbable templates is challenging. Macroporous templates have been employed in the assembly of chitosan materials with high surface area for wound healing^18^ and other applications^19,20^, however, nanoscale features were not observed in these examples, even when nanostructured templates were employed, such as oxidized nanofibrillar cellulose^21^. As the self-assembly of chitosan has been demonstrated to be heavily dependent upon ionic interactions^22^, it was hypothesized that a porous framework capable of such interactions may enable the nanoscale assembly of chitosan within its interstices.

In this work, hydrolytically-degradable, macroporous saccharide hydrogels are targeted as the sacrificial template. It is hypothesized that these organic frameworks containing sites for ionic interactions enable the loading and subsequent assembly of chitosan within the microstructured matrix, resulting in an easily-administered chitosan bandage with high surface area, and enhanced hemostatic efficiency. A macrocyclic oligosaccharide, β-cyclodextrin, serves as the primary building block of the hydrogel, and the hypothesized chemical compatibility between the saccharide-derived host and guest is expected to facilitate chitosan loading into the matrix. Cross-linking with a dianhydride is utilized to produce a polyester-linked porous gel capable of hydrolytic degradation under physiological conditions. The degradation of the prepared cyclodextrin polyester (CDPE) hydrogels is studied by proton nuclear magnetic resonance (^1^H NMR) spectroscopy, with assembly of chitosan within the matrix evaluated by scanning electron microscopy (SEM), following chitosan loading into the hydrogels and template removal. The hemostatic efficiency and biocompatibility of the composite chitosan-loaded CDPE (CDPE-Cs) hydrogels are then examined in vivo, in several animal models.

## Results

### Synthesis and characterization of hydrogels

CDPE networks were readily formed by the reaction between β-cyclodextrin and a dianhydride linker (3 equiv.) in dimethylsulfoxide (DMSO), when in the presence of an organic base (triethylamine, 3 equiv.) (Supplementary Fig. 1a). Reagents were combined in a flask before transfer into shallow, cylindrical molds. Following a short heating period at 70 °C to induce gelation, the resulting transparent gel was removed and cut to desired shapes with a razor blade. Several dianhydrides were screened, and 3,3′,4,4′-biphenyltetracarboxylic dianhydride (BPDA) was found to offer the greatest compatibility with the mold-casting process. Dianhydrides, such as pyromellitic dianhydride (PMDA), with greater solubility in the solvent system used induced a rapid increase in viscosity, causing difficulty during transfer to the molds. Gels were formulated at 16 wt%, calculated by the mass of solids as a fraction of the total mass. Gels with lower weight fractions were expected to yield greater porosities and, therefore, larger chitosan loading capacities, though at the cost of increasing fragility. However, at weight fractions below 14 wt%, gelation did not occur. Therefore, 16 wt% was chosen to provide gels that could be easily formed and handled, while facilitating the desired chitosan loading behavior.

**Fig. 1:**
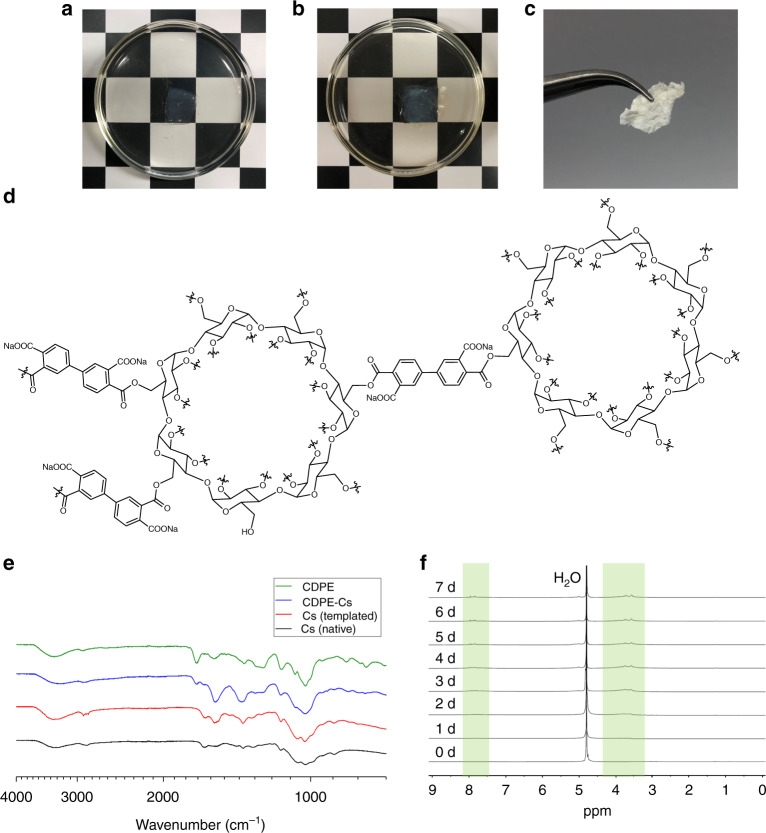
CDPE hydrogels are capable of loading chitosan, and degrade in alkaline conditions. **a** Image of CDPE gel, upon initial immersion in a 1 wt% chitosan solution. **b** Image of CDPE gel, after 7 days immersion in a 1 wt% chitosan solution, showing a slight increase in opacity of the gel. **c** Image of templated chitosan monolith, after lyophilization of composite gel and rinsing with alkaline solution to remove the CDPE template. **d** Simplified representation of the chemical structure of CDPE, indicating the possibility of diverse linkage regiochemistry. **e** FTIR spectra of materials at each stage of the templating process. **f** ^1^H NMR (500 MHz, in PBS D_2_O solution) spectra of the CDPE template at 24 h time points, showing degradation over 7 days

The gels were thoroughly washed with DMSO to remove soluble, insufficiently-crosslinked components and remaining reagents, followed by exchange into aqueous sodium chloride solution to convert ammonium carboxylate groups to their respective sodium salts. The gels were then exchanged into and stored in water. The resulting hydrogels could then be further used in their hydrated form, or dried by lyophilization for characterization. CDPE displayed stability when stored in hydrated form over a period of 2 weeks, however, full dissolution was observed in a concentrated potassium hydroxide (KOH) aqueous solution within ca. 1 min, confirming the base-sensitivity of the ester linkages, as designed. When lyophilized and placed in an excess of phosphate-buffered saline (PBS) at 37 °C, the gel degraded into water-soluble byproducts over the course of 7 days, as monitored by ^1^H NMR spectroscopy (Fig. 1f, Supplementary Fig. 2d, e). Turbidity measurements of the resulting solution confirmed solution clarity by full transmittance at 450 nm, and dynamic light scattering (DLS) measurements indicated the degradation of CDPE into a narrow distribution of small particles, with diameters centered around ca. 2.7 nm (Supplementary Fig. 2f).

**Fig. 2:**
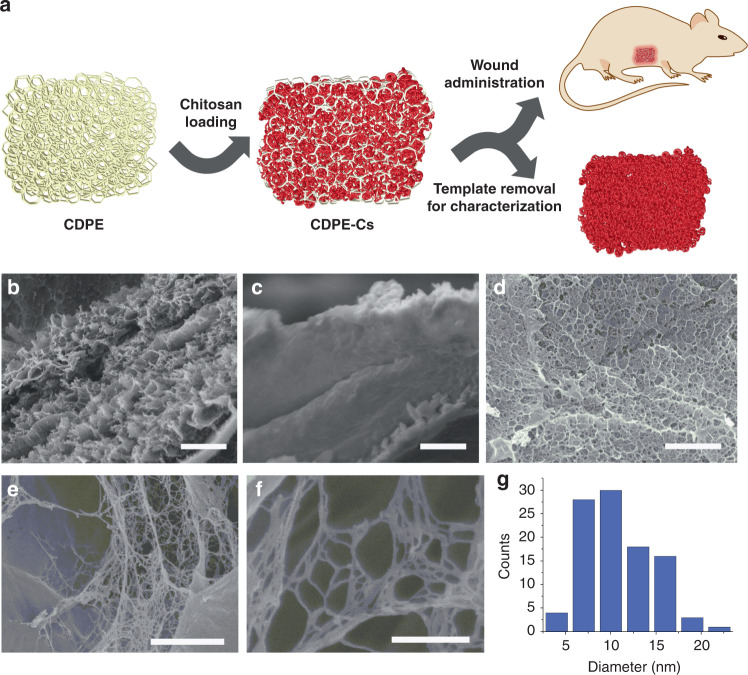
Prepared materials exhibit amorphous, macroporous morphology. **a** Simplified illustration of the chitosan templating process, administration of CDPE-Cs to wound site, and template removal for imaging. **b** SEM image of CDPE after drying by lyophilization, showing complex, porous morphology (scale bar is 2 µm). **c** SEM image of chitosan-loaded composite CDPE-Cs gel after lyophilization (scale bar is 2 µm). **d** SEM image of templated chitosan material, displaying a honeycomb-like structure (scale bar is 2 µm). **e**–**f** Magnified SEM images of nanofibrillar domains in the templated chitosan, with a web-like morphology within the network cavities (scale bars are 500 nm and 300 nm, respectively). **g** Histogram plot showing the distribution of fiber diameters measured by SEM

When viewed by SEM, CDPE exhibited a porous, complex morphology (Fig. 2b). To examine the ability of these CDPE templates to load and assemble chitosan, samples were submerged in a 1 wt% solution of chitosan (Fig. 1a), prepared in 1% aqueous acetic acid with 5 M aqueous sodium hydroxide added dropwise until a pH of 5.5 was reached. The pH of the chitosan solution was adjusted to a pH of 5.5, to more closely resemble physiological pH, with more alkaline conditions resulting in the precipitation of chitosan from solution. Over the course of 7 days, chitosan was allowed to migrate into the gel, and the samples gradually became slightly less transparent (Fig. 1b). CDPE templates displayed stability in the chitosan solution, remaining intact for the duration of the loading process, and were durable and malleable, facilitating ease of handling and subsequent administration. Control studies indicated only minor degradation of the template in 1% acetic acid over 7 days, as observed by ^1^H NMR spectroscopy (Supplementary Fig. 2c, e).

To probe the morphology of the loaded chitosan, the composite CDPE-Cs gel was lyophilized and rinsed with 5 M aqueous sodium hydroxide solution to remove the sacrificial CDPE template, yielding a monolithic mat of chitosan (Fig. 1c). The mass loss during template removal was ca. 81%, suggesting that the templated chitosan mat represented ca. 19% of the dry mass of the CDPE-Cs composite gel. The resulting templated chitosan monoliths were sufficiently durable to enable handling without fracturing, and were stable in water. However, the material could be fully dissolved in trifluoroacetic acid, with sonication. The physical structure of the templated chitosan mat was probed by SEM, revealing a porous, sponge-like material of highly-entangled fibers (Fig. 2d–f), with diameters of 9.2 ± 3.7 nm, presented as the mean ± standard deviation (SD) (*n* = 100) (Fig. 2g).

The CDPE template, loaded CDPE-Cs hydrogel, and templated chitosan after template removal were characterized individually by both solution- and solid-state techniques to probe their chemical compositions. Absorption at 1710 and 1603 cm^−1^ in the infrared (IR) absorption spectrum of the CDPE template confirmed the presence of ester linkages and carboxylate groups, respectively (Fig. 1e). The characteristic backbone vibration of β-cyclodextrin at 1032 cm^−1^ was also observed, indicating the integrity of the macrocyclic backbone of cyclodextrin was maintained. Upon chitosan loading into the template, the appearance of absorption bands at 2853, 1653, and 894 cm^−1^, corresponding to chitosan, was observed. Upon template removal, it was revealed that the absorption intensity at 1571 cm^−1^, corresponding to the N–H stretch of the primary amines, was somewhat amplified when compared with native chitosan, which suggests further deacetylation of the amine groups. When examined by ^1^H NMR spectroscopy, the degree of deacetylation was determined to be 67%, by the integration of the corresponding signals^23^, confirming an increase from the 62% measured for the native chitosan used (Supplementary Fig. 2b). Powder X-ray diffraction (PXRD) data indicated the amorphous character of CDPE, and the complete disappearance of peaks seen in the β-cyclodextrin starting material suggest the lack of remaining unreacted β-cyclodextrin crystals entombed in the matrix (Supplementary Fig. 3b). The templated chitosan material was also found to be predominantly amorphous by PXRD (Supplementary Fig. 3c).

### In vivo performance of CDPE-Cs hydrogels

The isolated chitosan mats were solid and non-malleable, which caused difficulty with application to the wound site. Therefore, we instead targeted the CDPE-Cs composite hydrogel bandages for study, as they were easily cut to shape, and conformed well to the injury sites when applied. The hemostatic efficiency of the chitosan-loaded hydrogels was evaluated in vivo, in acute liver punch models of rats, rabbits, and pigs. Though this model represents a non-lethal and rather modest injury to the liver, and thus cannot be generalized to all forms of bleeding from other sites, it allows comparison with other hemostatic dressings evaluated in this manner^9,15,21,24^. Liver punches of 1 cm diameters and 0.3 cm depths were excised in the animals using a circular blade fixed onto a syringe, to ensure the consistency of the induced bleeding across various animals. The circular blade-fixed syringe was used to cut the hemostatic hydrogels and the controls into the same dimensions (i.e., 1 cm × 0.3 cm) of the injury sites. The controls included conventional gauze, blank CDPE hydrogels, Surgicel® and Curaspon®. Surgicel® and Curaspon® are commercially available absorbable hemostatic agents mainly composed of oxidized regenerated cellulose and gelatin, respectively. After injury and excision of the standardized liver tissue, initial bleeding was absorbed into a conventional wound dressing. Then, various dressings were implanted into the injury sites and checked every 30 s for bleeding. The weights of the dressings were recorded before application and at the end of the experiments to measure the amounts of blood loss. A heparinized rabbit model was utilized at a certain dose of heparin (i.e., 250 IU/kg) to achieve bleeding that would not stop spontaneously after the liver injury. Mean arterial blood pressure (MAP) was recorded, only in rabbits, via insertion of a 20-gauge catheter into the right carotid artery and continuous recording of MAP under general anesthesia before the liver injury and for 1 h after the injury. Blood pressure was monitored on a polygraph using a Universal Oscillograph instrument. The animals were alive for the duration of the experiment.

**Fig. 3:**
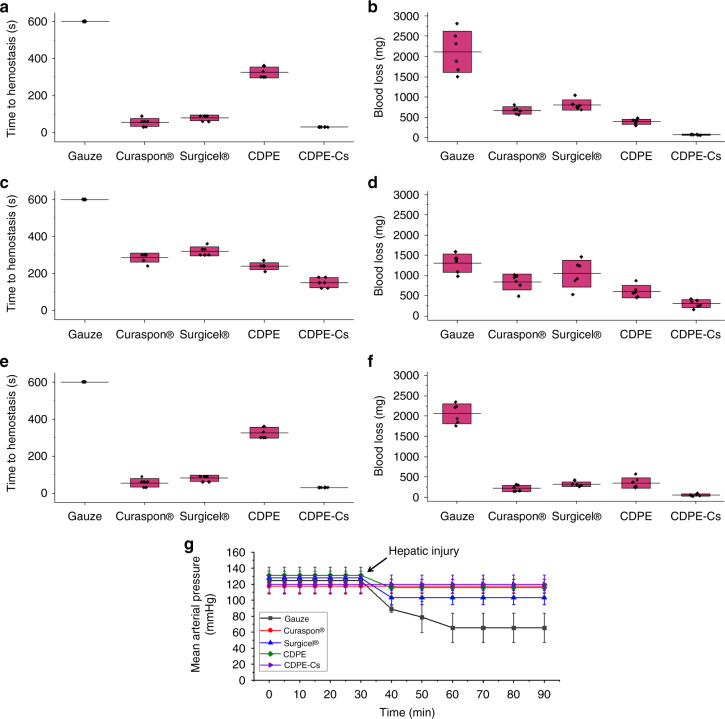
In vivo examination of CDPE-Cs hydrogels against several controls. **a** Conventional gauze dressings, Curaspon®, Surgicel®, and CDPE and CDPE-Cs hydrogels were applied immediately after induction of the liver injury and absorption of the initial bleeding from the injury sites, to determine the total time to hemostasis in rats (*n* = 6 animals per group). For gauze dressings, bleeding did not stop until the end of experiments (600 s). **b** The total amount of blood loss, measured over 10 min, in rats (*n* = 6 animals per group). **c** Time to hemostasis for rabbits (*n* = 6 animals per group). **d** Blood loss in rabbits (*n* = 6 animals per group). **e** Time to hemostasis for pigs (*n* = 6 animals per group). **f** Blood loss in pigs (*n* = 6 animals per group). **g** The mean arterial pressure of the rabbits were monitored over 90 min, where the measurements over the first 30 min represent the mean arterial pressure of animals before induction of the liver injury (*n* = 3 animals per group). Box plots correspond to means (center line) ± SD (boundaries). Source data are provided as a Source Data file

After application of conventional gauze to the injury sites, bleeding did not stop until the end of the experiments (10 min) in rats, rabbits, and pigs (Fig. 3a–e). The amounts of blood loss were also the most significant from injured livers treated with the conventional gauze dressings in rats, rabbits and pigs (Fig. 3b–f). Furthermore, the heparinized rabbits treated with conventional gauze failed to maintain a normal MAP (Fig. 3g). All the other groups maintained a normal MAP through the duration of the experiments, with slight decreases in the MAP observed for the Surgicel® and blank CDPE hydrogel groups after the liver injury. Generally, the absorbable dressings resulted in significantly faster hemostasis and lower amounts of blood loss compared to the conventional gauze dressings, although the experimental factors were fixed, except for the type of the dressing. In rats, significantly faster hemostasis was observed in the group treated with the CDPE-Cs hydrogel as compared to Surgicel®, Curaspon®, and the blank CDPE hydrogel (one-way ANOVA with Tukey’s multiple comparison test, *p* *<* 0.001). Similarly, significantly lower amounts of blood loss were also found in the group treated with the CDPE-Cs hydrogel as compared to Surgicel® (one-way ANOVA with Tukey’s multiple comparison test, *p* *<* 0.01) and Curaspon® (one-way ANOVA with Tukey’s multiple comparison test, *p* *<* 0.05). A similar pattern was also observed in rabbits, where the CDPE-Cs hydrogel resulted in shorter time to hemostasis (one-way ANOVA with Tukey’s multiple comparison test, *p* *<* 0.001), as compared to Surgicel®, Curaspon®, and the blank CDPE hydrogel, and a lower amount of blood loss than the amounts from rabbits treated with Surgicel® (one-way ANOVA with Tukey’s multiple comparison test, *p* *<* 0.001) and Curaspon® (one-way ANOVA with Tukey’s multiple comparison test, *p* *<* 0.05). However, it was observed that there were increases in the time to hemostasis and amounts of blood loss as compared to the rat and pig models, which might be due to the anticoagulant effect of the heparin utilized in the rabbit models. In pigs, there were significantly faster times to hemostasis (one-way ANOVA with Tukey’s multiple comparison test, *p* *<* 0.01 and *p* *<* 0.001) and lower amounts of blood loss (one-way ANOVA with Tukey’s multiple comparison test, *p* *<* 0.05) in the CDPE-Cs hydrogel group, as compared to the Surgicel® and the blank CDPE hydrogel groups, respectively. However, the differences in time to hemostasis and amounts of blood loss were not significant, when compared to Curaspon®, but in favor of the CDPE-Cs hydrogel treated group.

Preliminary evaluations of the biocompatibility and degradability of the dressings were performed via implantation of the dressings into the injured livers of rats. Photographs of the livers from rats following implantation of the different hemostatic dressings for 7 days and control untreated rats are illustrated in Supplementary Fig. 4. The active bleeding can be clearly seen in the animals immediately after the injury and after the application of the conventional gauze, whereas hemostasis is achieved after treatment with Curaspon®, Surgicel®, and CDPE-Cs hydrogels. After one week of implantation, visual and histological examinations of the tissues surrounding the livers were performed (Supplementary Fig. 5). Complete healing was observed in the groups treated with Curaspon® and CDPE-Cs hydrogels, while a small cut was observed in the Surgicel® group. No residues were found for Curaspon®, Surgicel®, or CDPE and CDPE-Cs hydrogels after one week of implantation. Necrosis and tissue damage could be observed only in the group treated with the conventional gauze dressings, and as expected, the conventional gauze remained intact after one week of implantation. Normal histological structures were observed in the groups, except for the livers implanted with the conventional gauze dressings and blank CDPE hydrogels, where significant inflammation was observed (Supplementary Fig. 5).

## Discussion

This work outlined the synthesis of a hyperbranched polyester hydrogel derived from β-cyclodextrin and its ability to load and assemble dissolved chitosan within its highly complex, porous network. We established the ability of the CDPE templates to degrade under physiological conditions, and further demonstrated the bioabsorbability and hemostatic efficacy of the chitosan-loaded (CDPE-Cs) composite hydrogels, when used as wound dressings, in acute liver punch models in rats, rabbits, and pigs. Significantly shorter times to hemostasis and reduced blood loss were observed in rats and rabbits, when compared to conventional gauze dressings and commercially available bioabsorbable dressings. The surface structure of the bandage is of critical importance, as the chitosan accelerates and strengthens blood clots through direct interaction with platelets and coagulation factors. Nanofibers offer the advantage of high surface area ratios^25^, and we hypothesized that the chitosan nanofibers dispersed within the CDPE-Cs hydrogels enabled robust interactions with these platelets and coagulation factors, increasing the pace and strength of the resulting clot^9,26^. Although, the hemostatic efficacy of chitosan, and the enhancement of this effect by increasing surface area, is well understood, the assembly of chitosan into the nanofibers observed in this work is likely due to a complex array of competing factors.

Due to the intricate interplay of competing forces, a wide range of microstructures have been observed by various chitosan assembly methods, with contributions from both complexation and aggregation^27^. Chitosan is only water-soluble in its polycationic form, where its deacetylated glucosamine groups must be sufficiently protonated to allow repulsive double-layer forces to overcome attractive forces^28^. It was, therefore, initially proposed that the assembly of aqueous chitosan solutions observed in the presence of certain anions was due to ionic complexation^29^. However, it was later determined that a physical gelation mechanism likely contributed to its assembly behavior, due to transfer of protons from chitosan to the anions, enabling the domination of attractive interchain forces^28^. It is expected that the assembly of chitosan within the confines of the microstructured CDPE template further convoluted its assembly behavior, and ultimately allowed access to structures presented in this work.

CDPE, itself, did not exhibit nanoscale features, and instead appeared as a porous matrix with micron-scale features (Fig. 2b). The templated chitosan did seem to exhibit a honeycomb-like appearance (Fig. 2d), which may be due to the packing of chitosan within the interstices of the CDPE network, however, the much smaller nanofibrillar features could not have been produced by physical imprinting alone. Chitosan nanofibers and nanofibrous mats have been previously reported^30,31^, but they are typically accessible by electrospinning, and the lowest average fiber diameters reported by this method, ca. 58 nm^32^, are still larger than the ca. 9.2 nm fibers obtained in this work. However, nanofibers of the closely related chitin have been produced with sub-10 nm diameters, merely by precipitation into a solvent that disrupts hydrogen bonding^33,34^, and this phenomenon may provide insight into the assembly behavior observed herein. To promote the physical assembly of chitosan chains by proton exchange and complexation phenomena, CDPE frameworks were constructed from dianhydride linkers to provide remaining carboxylate groups in the matrix sites to potentially interact with chitosan. When exchanged into water, the CDPE templates exhibited a tendency to curl slightly at the edges, despite being cast as flat, thick sheets. It was reasoned that this behavior was due to an increase in hydrogen bonding in the hydrogels, as although DMSO is a polar solvent, it does not contain an electropositive hydrogen to participate in hydrogen bonding as strongly as polar protic solvents, like water. When submerged in the chitosan solution, the CDPE hydrogels slowly uncurled to their original shape. Though some contribution to the shape restoration was likely due to the slight swelling induced by the loading of chitosan polymer chains into physical vacancies in the CDPE network, it is reasonable to expect this behavior to be, in part, due to the competition of chitosan with water for interaction with the CDPE network, which may disrupt hydrogen bonding with the solvent. Ionic exchange, or complexation, between the protonated amine groups of chitosan and carboxylate sites in the CDPE template may, then, enable the increase in attractive interchain forces of chitosan, conceivably enabling aggregation and the formation of the observed features. Therefore, although macroscopic features of the chitosan were attributed to physical templating by CDPE, the manifestation of nanofibrillar structures was ascribed to assembly of the chitosan chains within the interstices of the porous matrix, and was, correspondingly, only observed in discrete, confined regions of the network (Fig. 2e).

The breakdown of CDPE frameworks and CDPE-Cs composite hydrogels was investigated as a function of in vitro and in vivo conditions. Degradation of the CDPE framework proceeded under weakly or strongly alkaline conditions, through hydrolytic cleavage of the ester linkages between cyclodextrin moieties. Though ester hydrolysis can also proceed under acidic conditions, fragmentation of CDPE was not observed over 7 days when exposed to the mildly acidic chitosan solution, and only minor degradation of CDPE was observed by NMR spectroscopy and turbidity measurements in a 1% acetic acid solution over the same time period (Supplementary Fig. 2c, e). Derivatives of biphenyltetracarboxylic acid, the ring-opened form of the dianhydride linker, were formed as byproducts in the hydrolysis reaction, along with β-cyclodextrin derivatives (Supplementary Figs. 1b, 2d). The byproducts were soluble in PBS, used to emulate physiological conditions, with macroscopic CDPE breaking down to afford molecular-to-nanoscopic products within a clear solution after 7 days of exposure (Supplementary Fig. 2f). Although the analogous dissolution in PBS for CDPE-Cs composite hydrogels was not observed—even after 14 days—physiological degradability was observed during in vivo studies. Residual CDPE or CDPE-Cs hydrogels could not be observed after 1 week of implantation, likely indicating the importance of the in vivo microenvironment in providing for biodegradability vs. PBS hydrolytic stability in vitro. However, the lack of residues observed may be merely due to disintegration of the chitosan after degradation of the CDPE template, as chitosan may take up to 3 months to fully degrade in vivo^35^. In histopathological examinations of the tissue surrounding the injury sites in rats, no inflammation was observed for the CDPE-Cs hydrogel group, suggesting biocompatibility for the wound dressing (Supplementary Fig. 5).

In summary, the appearance of nanoscale features in chitosan mats by assembly within a β-cyclodextrin-derived hydrogel carrier template is reported. The assembled chitosan exhibited a honeycomb-like macroscopic morphology, and heavily entangled nanofibers, with diameters 9.2 ± 3.7 nm, were observed within the cavities of the structures. The CDPE carriers were designed to degrade hydrolytically under physiological conditions, and the efficacy of chitosan-loaded CDPE-Cs hydrogels as wound dressings in several animal models was demonstrated, in which shorter times to hemostasis and reduced blood loss were observed, in comparison to competitive dressing materials. These composite hydrogels are expected to provide a chitosan-based hemostatic technology, with a degradable delivery vessel, as biocompatible and bioabsorbable wound dressings for the reduction of blood loss in trauma scenarios. Future work will include testing the dressings in models of lethal hemorrhage to simulate clinical settings and to explore the clinical significance of the developed dressings.

## Methods

### Materials

Acetic acid (glacial), anhydrous dimethyl sulfoxide (DMSO), benzene, phosphate-buffered saline tablets, 3,3′,4,4′-biphenyltetracarboxylic dianhydride (BPDA), ethanol, methyl benzoate, heparin sodium, formalin solution (neutral buffered, 10%), triethylamine (Et_3_N), urethane, sodium chloride (NaCl), paraffin, deuterium oxide (D_2_O), acetic acid-d_4_, sodium hydroxide (NaOH), and chitosan (molecular weight 310–375 kDa) were purchased from Sigma-Aldrich and used as received. β-Cyclodextrin was obtained from TCI America and dried in vacuo at 70 °C for 12 h prior to use. Nanopure water (18.2 MΩ cm) was acquired from an EMD Millipore Milli-Q water filtration system.

### Characterization

Solution-state ^1^H and ^13^C NMR spectra were collected on a Varian Inova 500 spectrometer at room temperature. Solid-state cross-polarization magic angle spinning (CP-MAS) ^13^C NMR spectra were obtained on a Bruker Avance 400 spectrometer with a 4 mm rotor at a spin rate of 10.0 kHz. The instruments were interfaced to a LINUX computer using VNMR-J software, and spectra were processed with MestReNova version 9.0.1–13254 by Mestrelab Research S.L. Attenuated total reflection Fourier-transform infrared spectroscopy (ATR-FTIR) was performed on a Shimadzu IR-Prestige-21 spectrometer at a 1.0 cm^−1^ resolution. Thermogravimetric analysis (TGA) was performed on a Mettler-Toledo TGA 2 with a heating rate of 10 °C min^−1^ under argon atmosphere. Scanning electron microscopy (SEM) images were collected on a JEOL JSM-7500F FE-SEM equipped with a high brightness conical FE gun and a low aberration conical objective lens after platinum/palladium sputtering of the sample. Powder X-ray diffraction (PXRD) spectra were obtained on a Bruker D8 ADVANCE instrument with a Bruker LYNXEYE detector, with a 1 kW Cu X-ray tube, maintained at an operating current of 40 kV and 25 mA, operated in Bragg-Brentano para-focusing mode. Turbidity measurements were performed on a Shimadzu UV-2550 spectrophotometer at 450 nm with polystyrene cuvettes. Dynamic light scattering (DLS) measurements were performed on a Malvern Zetasizer Nano ZS with polystyrene cuvettes. Elemental analysis was performed at Midwest Microlab, LLC in Indianapolis, IN.

### CDPE hydrogel template synthesis

To a flame-dried round-bottom flask equipped with a stir bar was added β-cyclodextrin (1.750 g, 1.542 mmol), triethylamine (0.468 g, 4.62 mmol), and anhydrous dimethyl sulfoxide (14.42 mL). After dissolution, BPDA (1.361 g, 4.626 mmol) was added in one portion. The mixture was stirred for 5 min until the solids were evenly distributed throughout the mixture. The suspension was then poured into a shallow glass dish and heated to 70 °C on a hot plate for 2.5 h. After complete gelation, the system was allowed to return to room temperature. The gel was removed and cut to the desired shape with a razor blade and soaked in an excess of dimethyl sulfoxide for 1 h. The solvent was removed and exchanged with fresh dimethyl sulfoxide three additional times for 1 h each before placing the gel in an excess of water. The gel was then soaked in a saturated aqueous sodium chloride solution three times for 1 h each, before soaking three additional times in water for 1 h each. Samples were stored in water, or dried by lyophilization for characterization. ^13^C CP-MAS NMR (101 MHz, spin rate 10.0 kHz, ppm) *δ* 237.65–225.48, 179.16–160.74, 145.71–120.24, 105.51–92.82, 87.50–52.05. FTIR (cm^−1^): 3678–3015, 2927, 1776, 1710, 1603, 1576, 1363, 1289, 1245, 1145, 1077, 1032, 1002, 944, 845, 792, 769, 705. TGA: 25–90 °C, 6% mass loss; 90–235 °C, 4% mass loss; 235–280 °C, 29% mass loss; 280–330 °C, 11% mass loss; 330–390 °C, 14% mass loss; 390–465 °C, 8% mass loss; 465–500 °C, 10% mass loss; 18% mass remaining above 500 °C. Anal. calcd. for (C_6_H_10_O_5_)_1_·(C_14_H_12_O_8_Na)_6_·(H_2_O)_4_·(NaOH)_0.6_: C, 47.33; H, 4.00; N, 0.00; Na, 6.64. Found: C, 47.64; H, 4.23; N, 0.00; Na, 6.80.

### CDPE stability and degradation study

A phosphate-buffered saline tablet was dissolved in the corresponding amount of deuterated water to obtain a solution of 0.01 M phosphate buffer, 0.0027 M potassium chloride, and 0.137 M sodium chloride. A small, intact portion of lyophilized CDPE was cut to size by razor blade and placed in an NMR tube with the PBS D_2_O solution to obtain a concentration of 15.0 mg mL^−1^. The sample was then gently agitated at 37 °C (60 rpm), and analyzed by ^1^H NMR spectroscopy at 24 h intervals. After 7 days, the solution was transferred to a cuvette, and transmittance at 450 nm was measured to quantify turbidity, followed by dynamic light scattering measurements. A second sample was analogously prepared from lyophilized CDPE and 1% acetic acid in deuterated water, at a 15.0 mg mL^−1^ concentration, and analyzed by ^1^H NMR spectroscopy at 24 h intervals, with storage at room temperature without agitation.

### Chitosan loading to afford composite CDPE-Cs hydrogels

A chitosan solution was prepared by the dissolution of 5.000 g chitosan in 500.0 g acetic acid solution (1 wt% in nanopure water) to obtain a 1 wt% solution. After stirring for 24 h at room temperature, aqueous sodium hydroxide (5 M) solution was added dropwise, with vigorous stirring, until a pH of 5.5 was obtained. Pre-cut CDPE gels were submerged in the chitosan solution in a glass dish, and allowed to stand at room temperature for 7 days. Loaded gels were rinsed with water and lyophilized for characterization by IR spectroscopy and TGA. FTIR (cm^−1^): 3678–3015, 2927, 2853, 1710, 1653, 1576, 1363, 1295, 1245, 1154, 1077, 1032, 944, 894, 845, 769, 705. TGA: 25–135 °C, 12% mass loss; 205–360 °C, 43% mass loss; 360–500 °C, 12% mass loss; 33% mass remaining above 500 °C.

### CDPE template removal

Template removal was achieved by soaking lyophilized loaded gels in 100 mL aqueous NaOH solution (5 M) for 15 min, followed by rinsing with an excess of water. The resulting chitosan monolith was then dried in vacuo before characterization. The masses of the lyophilized CDPE-Cs composite gel and resulting dried templated chitosan mat were both measured to determine the mass loss during template removal, and to estimate the chitosan concentration in the composite material. For solution-state NMR spectroscopy of the templated chitosan, ca. 15 mg of the material was sonicated in deuterated trifluoroacetic acid until dissolved. ^1^H NMR (500 MHz, CF_3_COOD, ppm) *δ* 5.56–8.85 (m, 2H), 4.74–3.13 (m, 12H), 2.26–2.12 (m, 3H). ^13^C CP-MAS NMR (101 MHz, spin rate 10.0 kHz, ppm) *δ* 172.82, 110.78–95.86, 87.86–69.17, 66.02–55.74, 25.49–18.06. FTIR (cm^−1^): 3647–2997, 2920, 2853, 1647, 1571, 1471, 1417, 1378, 1319, 1153, 1065, 1030, 992, 947, 895, 805. TGA: 25–90 °C, 9% mass loss; 90–200 °C, 3% mass loss; 200–325 °C, 43% mass loss; 325–500 °C, 16% mass loss; 29% mass remaining above 500 °C.

### In vivo evaluation of CDPE-Cs hydrogels

Animals were purchased and kept at room temperature, and allowed food and water ad libitum. The protocol was approved by the Assiut University Animal Ethical Committee (Ethical approval number 1730030), and was in compliance with all relevant ethical regulations for animal testing and research. Animals were maintained under general anesthesia during the duration of experiments and euthanatized while under anesthesia at the end of the experiments.

Rats: The acute liver punch biopsy model was developed as described previously^24,36^ (with slight modifications, to evaluate the biocompatibility/biodegradation of the developed hemostatic hydrogel and to examine the hemostatic efficiency via measurements of the time to hemostasis and blood loss). Adult male Sprague-Dawley rats (ca. 300 g) were anaesthetized with an intraperitoneal injection of sodium thiopental (50 mg kg^−1^) and their abdominal hair was shaved. The rats were randomly selected from the laboratory population and divided into five groups (*n* = 6 animals per group). After opening the abdomen, the liver was exposed and an incision of ca. 1 cm in diameter, at an angle perpendicular to the tissue to a depth stop of ca. 0.3 cm, was produced on the liver. The tissue in the center of the injury site was removed using forceps and surgical scissors. Sterile gauze was used to absorb initial bleeding, and then, conventional gauze, Curaspon®, Surgicel®, CDPE and CDPE-Cs hydrogels were implanted into the injury site. A blade inserted onto a syringe was utilized to induce the injury, and to cut the gauze, sponge, and hemostatic hydrogels to match the dimensions of the wound (i.e., ca. 1 cm in diameter × 0.3 cm in depth). Several pieces were applied from the conventional gauze, Curaspon®, and Surgicel®, and bleeding was evaluated every 30 s for 10 min. CDPE and CDPE-Cs hydrogels were applied only once. The time to hemostasis and blood loss were measured. The hemostatic time was recorded as the last time of application where bleeding was not observed from the injury site. Weights of the dressings before and after applications were recorded to calculate the amount of blood loss. For hydrogels, blood loss was calculated based on the bleeding from the injury that was absorbed into the surrounding gauze.

For biocompatibility/biodegradation studies, the conventional gauze dressings, Curaspon®, Surgicel®, and CDPE and CDPE-Cs hydrogels were implanted and left inside the liver after the first application. The initial bleeding was absorbed with gauze. The abdomen was then closed with sutures, and the area was sterilized with povidone iodine. After one week, the sutures were removed and the liver was visually examined for wound healing. The animals were euthanized at the end of the experiments, and specimens from the liver tissues surrounding the injury sites were harvested for histological examination. Tissues were fixed immediately in 10% neutral formalin for 24 h, washed in running tap water for at least 2 h, and then immersed in 70% ethanol. Dehydration in ascending concentrations of ethanol (i.e., 70, 90, 95, and 100%) was carried out, followed by clearing the specimens in double embedding (1 g celloidin dissolved in 100 mL methyl benzoate, three changes for 3 days). The specimens were then washed in benzene (two changes, each for 15 min). Impregnation in paraffin was performed, followed by embedding the specimens in hard paraffin. Sections of 5 µm thickness were prepared using a rotary microtome (CUT 4050, microTec Laborgeräte GmbH, Walldorf, Germany). Specimens were stained with Hematoxylin and Eosin (H&E) stain for histological examination and imaged by Olympus BX53 light microscope (Olympus Co., Tokyo, Japan).

Rabbits: Adult male New Zealand white rabbits (ca. 2 kg) were kept in large cages and were anesthetized with intraperitoneal injection of urethane (1 g kg^−1^) prior to the surgical procedures. Rabbits were randomly selected from the laboratory population and divided into five groups (*n* = 6 animals per group) and treated exactly as mentioned in the in vivo rat section (vide supra). In a separate study, the MAP was monitored during the surgical procedures (*n* = 3 animals per group). A 20-gauge catheter was surgically cannulated into the right carotid artery and the MAP was recorded continuously under general anesthesia before the liver injury and for 1 h after the injury. Blood pressure was monitored on a polygraph using the Universal Oscillograph (Harvard Apparatus, South Natick, MA). A heparinized rabbit model was utilized at a certain dose of heparin (i.e., 250 IU kg^−1^) to achieve bleeding that would not stop spontaneously after the liver injury.

Pigs: Female *Sus scrofa domesticus* pigs (ca. 50 kg) were anesthetized with intraperitoneal injection of sodium thiopental (50 mg kg^−1^). Identical procedures were performed to evaluate the time to hemostasis and blood loss, as mentioned previously. Six pigs were utilized, and five incisions were performed in each one, as previously described, and conventional gauze, Curaspon®, Surgicel®, and CDPE and CDPE-Cs hydrogels were applied.

Values are presented as the mean ± SD of at least six independent experiments. Significant differences between groups were evaluated by one-way ANOVA followed by Tukey’s multiple comparison tests. A sample size of six animals per group, for time to hemostasis and blood loss experiments, was expected to provide a power of ca. 0.9, with a Type I error probability for rejection of the null hypothesis of 0.05. Differences between different groups were considered significant for *p* values less than 0.05.

### Reporting summary

Further information on research design is available in the Nature Research Reporting Summary linked to this article.

## Supplementary information

Supplementary Information

Peer Review File

Reporting Summary

## Source data

Source Data

## Data Availability

The source data underlying Fig. 3 is provided as a Source Data file. All other data supporting the conclusions of this manuscript are available from the corresponding authors upon request.
