# Author Correction: Absorbable hemostatic hydrogels comprising composites of sacrificial templates and honeycomb-like nanofibrous mats of chitosan

**DOI:** 10.1038/s41467-025-62683-0

**Published:** 2025-08-13

**Authors:** Eric E. Leonhardt, Nari Kang, Mostafa A. Hamad, Karen L. Wooley, Mahmoud Elsabahy

**Affiliations:** 1 Departments of Chemistry, Chemical Engineering, Materials Science & Engineering, and The Laboratory for Synthetic-Biologic Interactions, Texas A&M University, College Station, TX 77842-3012 USA; 2 Department of Surgery, Faculty of Medicine, Assiut University, Assiut, 71515 Egypt; 3 Department of Pharmaceutics and Assiut International Center of Nanomedicine, Al-Rajhy Liver Hospital, Assiut University, Assiut, 71515 Egypt; 4 Misr University for Science and Technology, 6th of October City, 12566 Giza, Egypt

Correction to: *Nature Communications* 10.1038/s41467-019-10290-1, published online 24 May 2019

In the version of the article initially published, due to a preparation error, the graph shown as Fig. 3a was an inadvertent duplicate of Fig. 3e. Due to the age of the paper, the figure cannot be replaced. Figure 3, below, serves to update the figure.

**Fig. 3:**
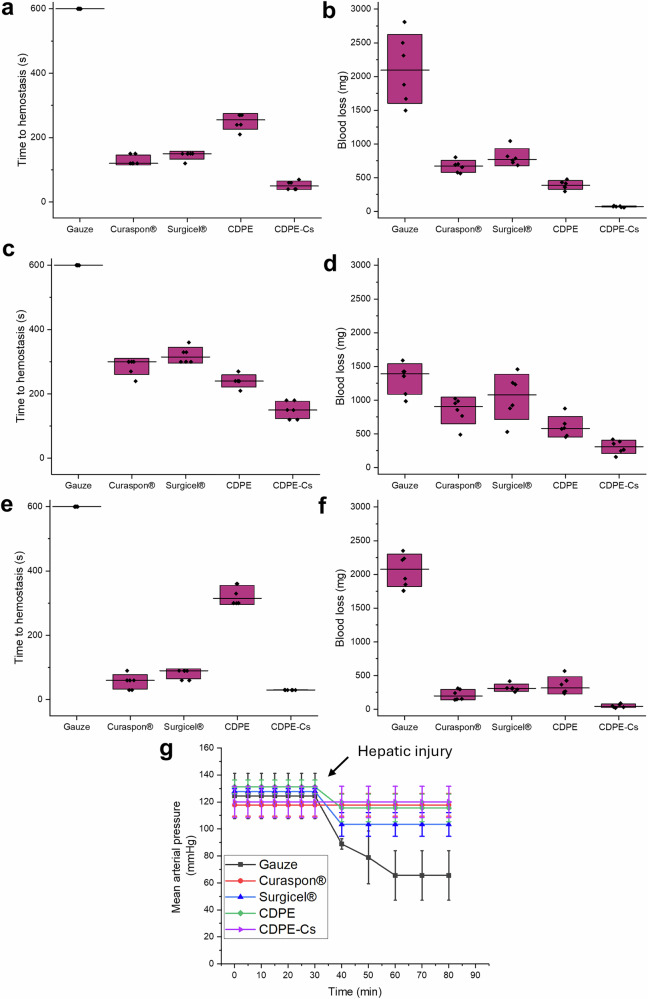
Corrected Fig. 3a

