# Supplementary material for: Absorbable hemostatic hydrogels comprising composites of sacrificial templates and honeycomb-like nanofibrous mats of chitosan
Source: Nat Commun. 2019 May 24;10:2307. doi: 10.1038/s41467-019-10290-1 (PMC6534699; doi:10.1038/s41467-019-10290-1)
Supplement: Supplementary file 1 — Supplementary Information [file 41467_2019_10290_MOESM1_ESM.pdf]

Supplementary Information for

Absorbable Hemostatic Hydrogels Comprising Composites of Sacrificial Templates and Honeycomb-like Nanofibrous Mats of Chitosan

Eric E. Leonhardt,[1] Nari Kang,[1]

Mostafa A. Hamad,[2] Karen L. Wooley,[1*] and Mahmoud Elsabahy[1,3*]

[1]Departments of Chemistry, Chemical Engineering, Materials Science & Engineering, and The Laboratory for Synthetic-Biologic Interactions, Texas A&M University, College Station, Texas 77842-3012, United States

[2]Department of Surgery, Faculty of Medicine, Assiut University, Assiut 71515, Egypt

[3]Department of Pharmaceutics and Assiut International Center of Nanomedicine, Al-Rajhy Liver Hospital, Assiut University, Assiut 71515, Egypt

*Correspondence:

wooley@chem.tamu.edu

mahmoud.elsabahy@chem.tamu.edu

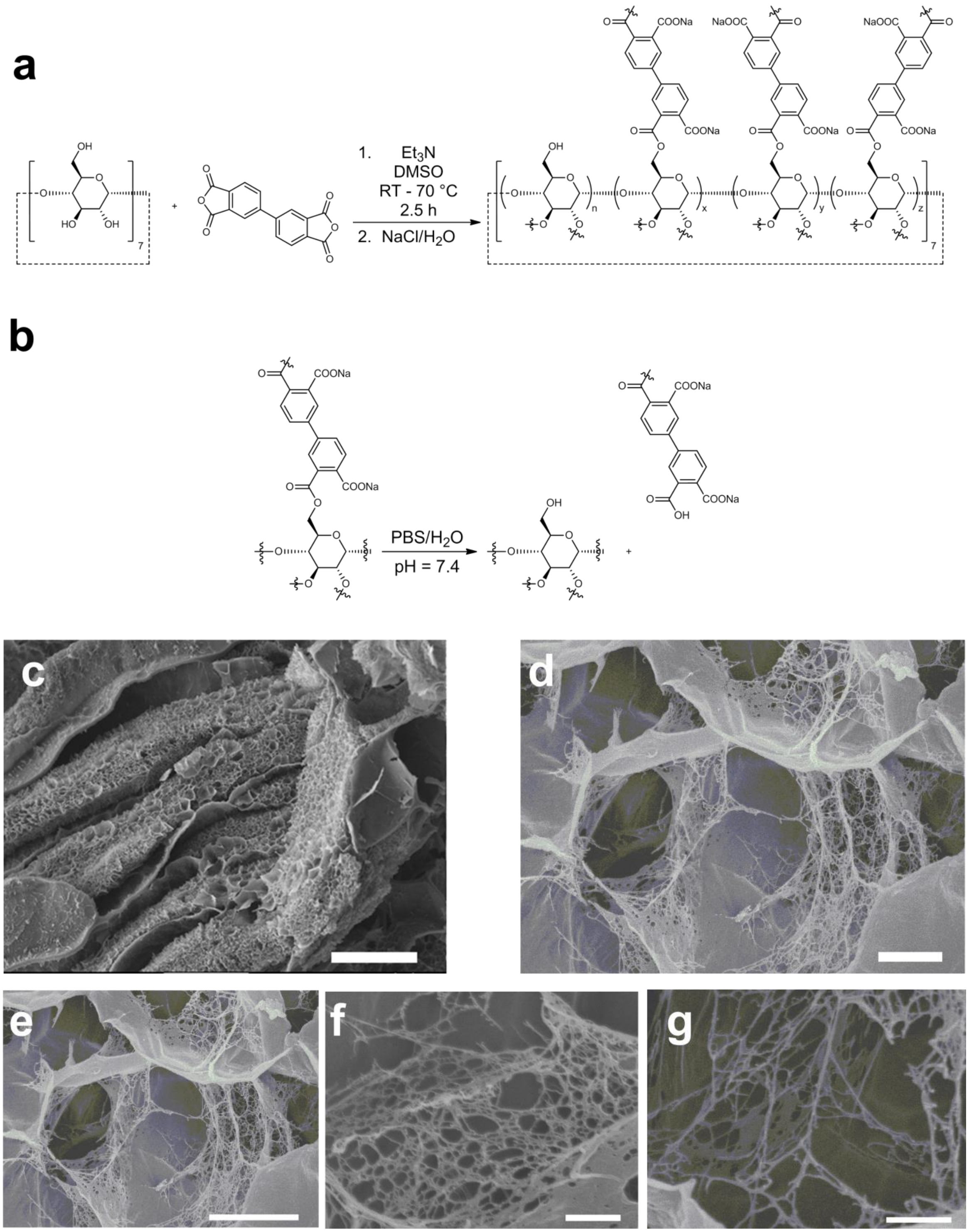

Supplementary Figure 1. Overview of cyclodextrin polyester (CDPE) synthesis and degradation. **a** Synthetic scheme detailing the production of CDPE hydrogels. **b** Potential ester hydrolysis over time, shown for a single CD repeat unit, yielding the ring-opened byproduct of the aromatic linker and regenerating the hydroxylic cyclodextrin moiety. **c,** SEM image of CDPE template after lyophilization (scale bar is 10 µm). **d–g** SEM images of the templated chitosan mat (scale bars are 1 µm, 2 µm, 500 nm, and 400 nm, respectively)

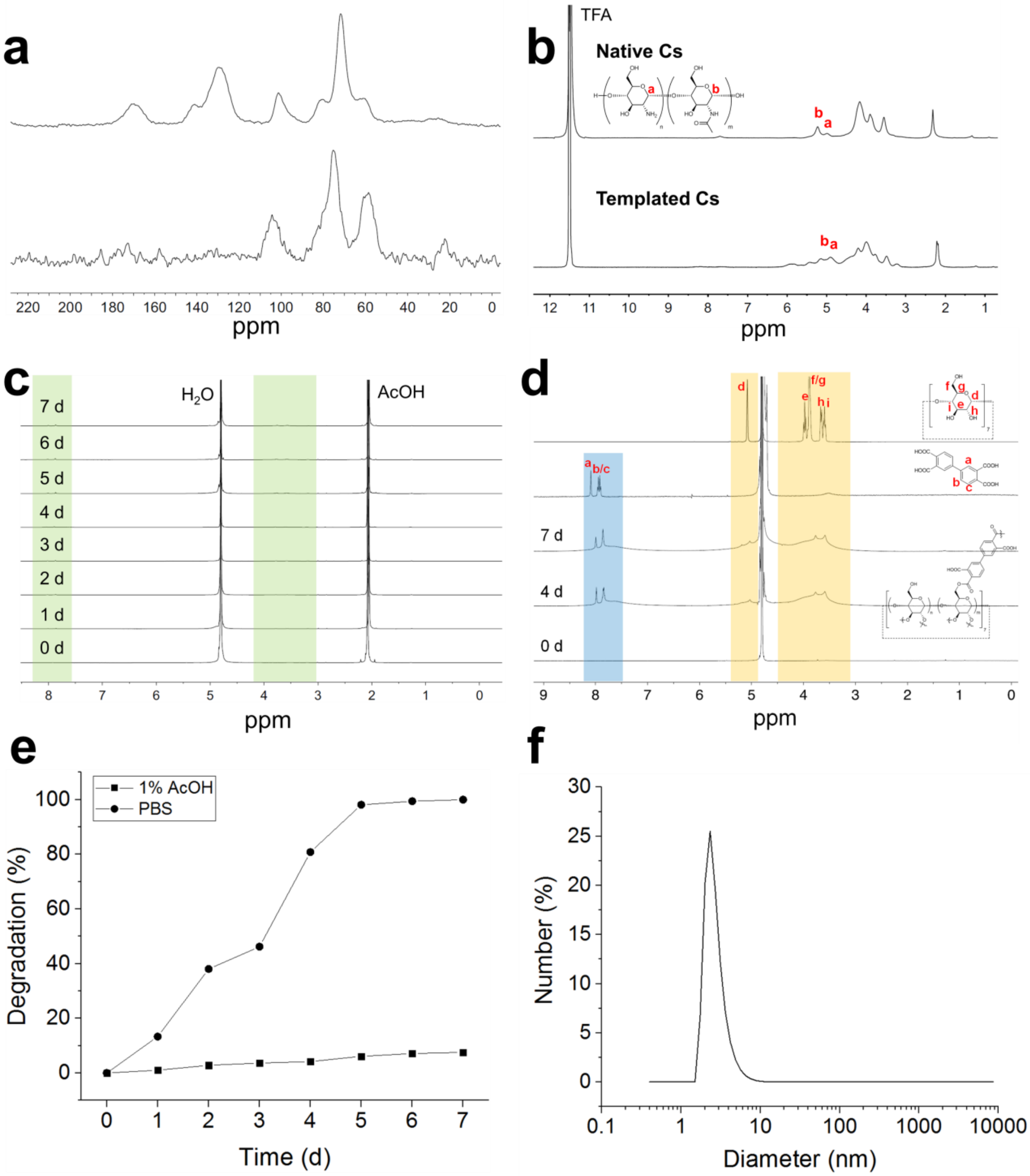

Supplementary Figure 2. NMR data and degradation studies for reported materials. **a** Solid-state CP-MAS ^{13}C NMR (101 MHz) spectrum of dried CDPE template (top) and templated chitosan material (bottom). **b** ^{1}H NMR (500 MHz) spectra of native and templated chitosan in deuterated trifluoroacetic acid. **c** ^{1}H NMR (500 MHz) spectra of CDPE template, in 1% acetic acid solution in D_2O, over 7 d. **d** ^{1}H NMR (500 MHz) spectra of CDPE template in D_2O at selected time points (lower three spectra), showing degradation into derivatives of the corresponding byproducts (upper two spectra). **e** Degradation of CDPE template in PBS and 1% AcOH deuterated aqueous solutions, as measured by the cumulative signal increase observed by ^{1}H NMR spectroscopy. For the sample in 1% AcOH, the degree of degradation was calculated using the acetic acid peak as an internal reference. For the sample in PBS, the degree of degradation was normalized to the signal integration at 7 d. **f** Particle size distribution of CDPE template solution in PBS after 7 d, measured by dynamic light scattering (DLS)

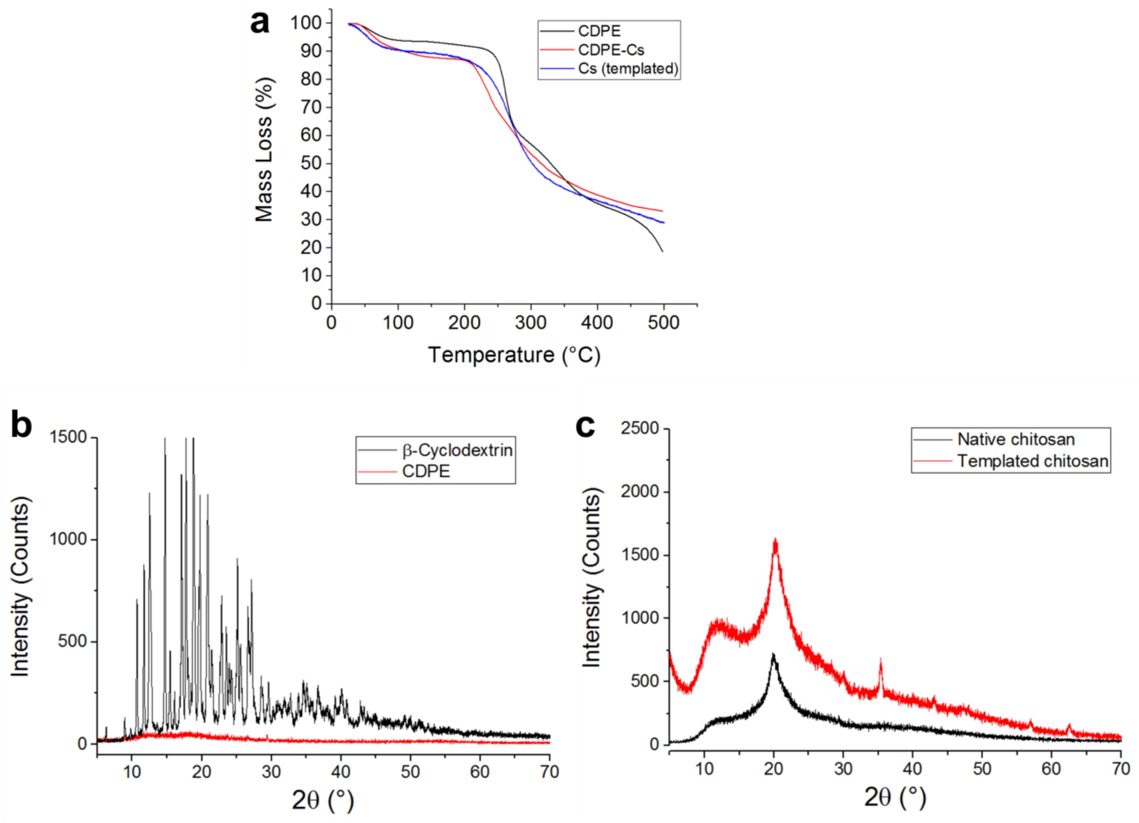

Supplementary Figure 3. Thermogravimetric analysis (TGA) and powder X-ray diffraction (PXRD) data. **a** TGA curves for CDPE template, chitosan-loaded hydrogel (CDPE-Cs), and templated chitosan after template removal. **b** PXRD spectra of β-cyclodextrin and CDPE, confirming the amorphous morphology of the gel. **c** PXRD spectra of native chitosan and the templated chitosan material, indicating a predominantly amorphous character of the materials

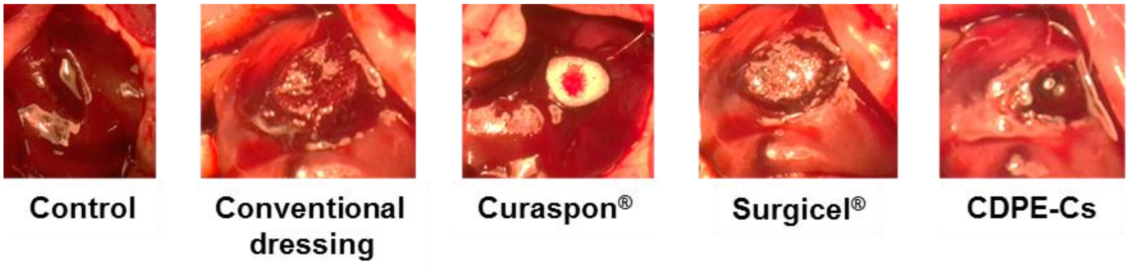

Supplementary Figure 4. Images of the livers from rats after treatment with selected hemostatic dressings and control untreated animals. Conventional gauze dressing, Curaspon®, Surgicel®, and cyclodextrin-chitosan (CDPE-Cs) hydrogels were applied immediately after the gauze-based absorption of the initial bleeding from the injury sites

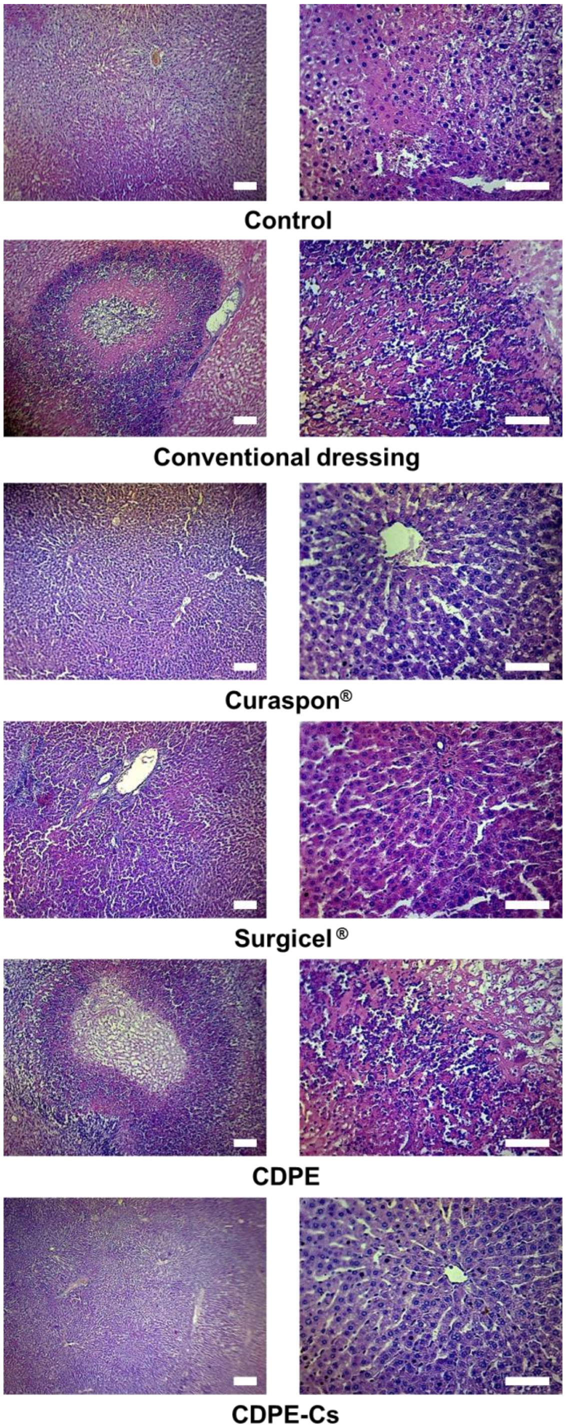

Supplementary Figure 5. Photomicrographs of the histological structures of the livers harvested from rats after treatment with selected hemostatic dressings (H & E stain, scale bars for images on the left are 100 µm and scale bars for images on the right are 50 µm). Conventional dressing, Curaspon®, Surgicel®, CDPE, and CDPE-Cs were applied immediately after the gauze-based absorption of the initial bleeding from the injury sites. One week after the implantation of the various hemostatic agents, the tissues surrounding the livers were examined
