# Supplementary material for: Absorbable hemostatic hydrogels comprising composites of sacrificial templates and honeycomb-like nanofibrous mats of chitosan
Source: Nat Commun. 2019 May 24;10:2307. doi: 10.1038/s41467-019-10290-1 (PMC6534699; doi:10.1038/s41467-019-10290-1)
Supplement: Supplementary file 2 — Peer Review File [file 41467_2019_10290_MOESM2_ESM.pdf]

Reviewers' comments:

Reviewer #1 (Remarks to the Author):

In this manuscript, the authors presented chitosan-based absorbable hemostatic honeycomb-like nanofibrous mats via using beta-CD hydrogels sacrificial templates. The materials show high hemostatic efficiency and biocompatibility in vivo in rats, rabbits and pigs even compared to conventional gauze dressings and commercial absorbable hemostatic dressings, Surgicel® and Curaspon® dressings. However, first, the biocompatibility and absorbable hemostatic agents and dressings have been commercial, just as the authors described in Introduction. Second, chitosan-based materials, including nanofibes mats, sponges were reported in lots of references, such as Biomaterials (2010, 31, 1270-1277), International Journal of Biological Macromolecules (2015, 75, 322-329) and Carbohydrate Polymers (2013, 97, 65-73). Although this honeycomb-like nanofibrous chitosan-based mats might possess some properties higher than those published chitosan based materials, the novelty and inspiration is so high to be published in Nat. Commun.
Others technical questions: (1) The mats-like or sponges-like hemostatic materials are the best type in application? The wound is not regular and blood loss speed is not controlled. If the hemostatic materials are powders-like and form solid after absorption of blood, it may be also useful in some practical conditions? (2) What is the mechanical performance of chitosan-based absorbable hemostatic honeycomb-like nanofibrous mats? (3) The resolution of Fig. 3 should be improved.

Reviewer #2 (Remarks to the Author):

Chitosan has been intensively investigated for hemostatic applications, and many chitosan-based hemostats for top dressings have been commercialized. In this manuscript, the authors creatively developed absorbable chitosan nanofiber-loaded cyclodextrin polyester (CDPE-Cs) hydrogels for effective hemostasis in vivo. They showed shorter hemostatic time and less blood loss compared to some other absorbable hemostats such as Surgicel® and Curaspon®. Furthermore, they were degraded in 7 days in the liver and caused no necrosis and tissue damage. This hemostatic hydrogel are good to the wounded persons because they are biodegradable in the physiological conditions and no removal should be carried out. This is helpful for reducing pains and medical costs. Such biodegradable cyclodextrin polyesters and CDPE-Cs may also find other important biomedical applications such as drug-carriers. Here are my concerns and suggestions which should be addressed.

1.p.4, The authors agree that the surface area including chitosan-based materials is a critical initiative for improving their effectiveness, so they developed a strategy to make chitosan nanofibers with extremely ultrafine diameters of 9.2 ± 3.7 nm, with an intention of utilizing these nanofibers for enhancing hemostatic performance. The results showed these nanofibers really gave great contributions. Please explain how the chitosan nanofibers embedded in the CDPE-Cs hydrogels contribute to the hemostasis. In other words, the hemostatic mechanism of the CDPE-Cs hydrogels should be discussed. In fact, the surface structure and morphology of the hemostats is also very important to its effectiveness, since they are the frontlines interacting with blood components to activate/aggregate blood cells, platelets, and clotting factors.
2.p.10, How about the morphology of the pore walls? Are they also composed of nanofibers? An SEM image with magnification between Fig. 2d and Fig. 2e would give information.
3.p.14, Fig. 3, (1). Why the blood loss on rat, rabbit, and pig wounds is same? (2). A supplemental table summarizing the time to hemostasis and blood loss would give clearer comparison. (3). How about the hemostatic performance of the templated chitosan?
4.p.19, The in vivo biodegradation of chitosan takes more than 3 months (Biomaterials 1997, 16, 567). Since the dissolution in PBS for CDPE-Cs composite hydrogels was not observed—even after 14 d—physiological degradability was observed during in vivo studies, is it possible that the in vivo degradability of CSPE-Cs is disintegration after the hydrolytic degradation of CDPE frame?

5.p.20, the molecular weight and degree of deacetylation of CS should be determined, since they may affect the in vivo biodegradation.
6.p.23, Why the assemble of chitosan in the CDPE hydrogels should be in pH=5.5? Whether chitosan flocculations are formed at this pH? What is the mass ratio of CS in the CDPE-Cs hydrogels?
7.SPI Fig. 4., the red background with dark-red liver and red blood makes the images poor contrast.

Reviewer #3 (Remarks to the Author):

As a trauma surgeon, I am limiting my comments to the animal models and the hemostatic data. The liver punch injury is a fairy minimal injury that generates low pressure bleeding. This is very different from high flow/high pressure bleeding such as seen in vascular injuries. So, one of the limitations that should be acknowledged is that the data are from a non-lethal, and rather modest injury to the liver, and this can not be generalized to all forms of bleeding from other sites.

Similarly, the bleeding time and volume of blood lost (Figure 3) in all the three animal models may have been statistically different between the various hemostatic agents, but clinically these differences were rather meaningless. For example, in a pig blood loss of 50, 100 or 200 mg despite a four fold difference is clinically insignificant.

In clinical practice surgeons often use electrocautry or other similar tools to contol liver bleeding rather than using hemostatic agents. This also makes the model rather artificial.

Would this product work in a rapidly bleeding model of lethal hemorrhage?

Point-by-point response to the reviewers

Reviewer # 1

Remarks to the author:
In this manuscript, the authors presented chitosan-based absorbable hemostatic honeycomb-like nanofibrous mats via using beta-CD hydrogels sacrificial templates. The materials show high hemostatic efficiency and biocompatibility in vivo in rats, rabbits and pigs even compared to conventional gauze dressings and commercial absorbable hemostatic dressings, Surgicel® and Curaspon® dressings. However, first, the biocompatibility and absorbable hemostatic agents and dressings have been commercial, just as the authors described in Introduction. Second, chitosan-based materials, including nanofibers mats, sponges were reported in lots of references, such as Biomaterials (2010, 31, 1270-1277), International Journal of Biological Macromolecules (2015, 75, 322-329) and Carbohydrate Polymers (2013, 97, 65-73). Although this honeycomb-like nanofibrous chitosan-based mats might possess some properties higher than those published chitosan based materials, the novelty and inspiration is so high to be published in *Nat. Commun*.

Others technical questions:
1. The mats-like or sponges-like hemostatic materials are the best type in application? The wound is not regular and blood loss speed is not controlled. If the hemostatic materials are powders-like and form solid after absorption of blood, it may be also useful in some practical conditions?

We desired malleable dressings (e.g. gels, fabrics, etc.) for their ability to conform to the injury site. Powdered hemostats, although they are easy to use, with minimal preparation time, may have a tendency to float on the bleeding surface, compromising their hemostatic efficiency (e.g. ACS Biomater. Sci. Eng. 2017, 3, 3675). Furthermore, the precise application of powdered hemostatic materials to the bleeding site can be more difficult, with the increased possibility of dispersing the powder outside of the targeted area. Therefore, we targeted the composite hydrogel bandages in this study for their improved administration.

2. What is the mechanical performance of chitosan-based absorbable hemostatic honeycomb-like nanofibrous mats?

Both the CDPE-Cs composites and isolated Cs mats were sufficiently robust to enable handling for the studies performed. Comments regarding our observations of the mechanical properties of these materials have been expanded in the revised manuscript (pp. 8 and 12).

3. The resolution of Fig. 3 should be improved.

The resolution has been improved, and a table summarizing the results of Fig. 3 is included to improve the legibility of this figure.

We thank the reviewer for these helpful comments.

Reviewer # 2

Remarks to the author:
Chitosan has been intensively investigated for hemostatic applications, and many chitosan-based hemostats for top dressings have been commercialized. In this manuscript, the authors creatively developed absorbable chitosan nanofiber-loaded cyclodextrin polyester (CDPE-Cs) hydrogels for effective hemostasis in vivo. They showed shorter hemostatic time and less blood loss compared to some other absorbable hemostats such as Surgicel® and Curaspon®. Furthermore, they were degraded in 7 days in the liver and caused no necrosis and tissue damage. This hemostatic hydrogel are good to the wounded persons because they are biodegradable in the physiological conditions and no removal should be carried out. This is helpful for reducing pains and medical costs. Such biodegradable cyclodextrin polyesters and CDPE-Cs may also find other important biomedical applications such as drug-carriers. Here are my concerns and suggestions which should be addressed.

1. p.4, The authors agree that the surface area including chitosan-based materials is a critical initiative for improving their effectiveness, so they developed a strategy to make chitosan nanofibers with extremely ultrafine diameters of 9.2 ± 3.7 nm, with an intention of utilizing these nanofibers for enhancing hemostatic performance. The results showed these nanofibers really gave great contributions. Please explain how the chitosan nanofibers embedded in the CDPE-Cs hydrogels contribute to the hemostasis. In other words, the hemostatic mechanism of the CDPE-Cs hydrogels should be discussed. In fact, the surface structure and morphology of the hemostats is also very important to its effectiveness, since they are the frontlines interacting with blood components to activate/aggregate blood cells, platelets, and clotting factors.

As the reviewer stated, the surface structure of the bandage is of critical importance, as the chitosan accelerates and strengthens blood clots through direct interaction with platelets and coagulation factors. Nanofibers offer the advantage of high surface area ratios (e.g. Mar. Drugs, ***2018****, 16, 273), and we hypothesized that the chitosan nanofibers dispersed within the CDPE-Cs hydrogels enabled robust interactions with these platelets and coagulation factors, increasing the pace and strength of the resulting clot (Adv. Mater.,* ***2018****, 30, 1700859 and Biomaterials,* ***2011****, 32, 6655). In the revised manuscript (p. 17), we have highlighted the hypothesized effects of chitosan nanofibers in these hemostatic materials.*

2. p.10, How about the morphology of the pore walls? Are they also composed of nanofibers? An SEM image with magnification between Fig. 2d and Fig. 2e would give information.

The pore walls of the templated chitosan mats are not composed of nanofibers, but rather nanofibers exist within the smooth pore walls of the material. We have added an additional SEM image (Supplementary Figure 1d) that shows a magnification between Fig. 2d and Fig. 2e, in which the described features can be observed.

3. p.14, Fig. 3, (1). Why the blood loss on rat, rabbit, and pig wounds is same? (2). A supplemental table summarizing the time to hemostasis and blood loss would give clearer comparison.

For all animals, injuries of identical dimensions were induced, which may explain the reason for the similar blood loss. However, in error, Fig. 3d was a duplicate of Fig. 3b, and this has been corrected in the revised manuscript. We have also included a table summarizing the time to hemostasis and blood loss in Fig. 3 for increased clarity.

4. How about the hemostatic performance of the templated chitosan?

The isolated chitosan mats were solid and non-malleable, which caused difficulty with application to the wound site. Therefore, we instead targeted the CDPE-Cs composite hydrogel bandages, as they were easily cut to shape, and conformed well to the injury sites when applied. The manuscript has been modified accordingly to comment on the performance of the templated chitosan (p. 12).

5. p.19, The in vivo biodegradation of chitosan takes more than 3 months (Biomaterials 1997, 16, 567). Since the dissolution in PBS for CDPE-Cs composite hydrogels was not observed—even after 14 d—physiological degradability was observed during in vivo studies, is it possible that the in vivo degradability of CSPE-Cs is disintegration after the hydrolytic degradation of CDPE frame?

The reviewer is likely correct. We sincerely appreciate this comment and recommendation. The manuscript has been modified to include the fact that the observations in vivo may be due to disintegration of chitosan after the degradation of the CDPE carrier, and does not necessarily indicate total degradation of the materials (pp. 16 and 20).

6. p.20, the molecular weight and degree of deacetylation of CS should be determined, since they may affect the in vivo biodegradation.

The degree of deacetylation, determined by proton NMR spectroscopy (according to Lavertu, M. et al. J. Pharm. Biomed. Anal. ***2003****, 32, 1149) was 62% for the native chitosan and 67% for the templated chitosan. The molar mass of the chitosan used is 310 - 375 kDa. We have included this information in the revised manuscript (pp. 10–11 and 21).*

7.p.23, Why the assemble of chitosan in the CDPE hydrogels should be in pH=5.5? Whether chitosan flocculations are formed at this pH? What is the mass ratio of CS in the CDPE-Cs hydrogels?

It was our desire to maintain as close to physiological pH as possible for the wound dressings. A pH of 5.5 was found to be as close to physiological pH as we could obtain, as at greater alkalinity, chitosan flocculations began to form. We determined the mass composition of the wound dressings to be 19% chitosan, according to the (dry) mass change before and after template removal. We have included this information in the revised manuscript (pp. 8–9 and 25).

8. SPI Fig. 4., the red background with dark-red liver and red blood makes the images poor contrast.

The red background color is due to the abdomen tissues that surround the injury, which unfortunately caused great difficulty in providing clearer contrast. We have attempted to improve the contrast of the image by slightly increasing the image brightness.

We thank the reviewer for these helpful comments.

Reviewer # 3

Remarks to the author:
As a trauma surgeon, I am limiting my comments to the animal models and the hemostatic data. The liver punch injury is a fairy minimal injury that generates low pressure bleeding. This is very different from high flow/high pressure bleeding such as seen in vascular injuries. So, one of the limitations that should be acknowledged is that the data are from a non-lethal, and rather modest injury to the liver, and this cannot be generalized to all forms of bleeding from other sites.

Similarly, the bleeding time and volume of blood lost (Figure 3) in all the three animal models may have been statistically different between the various hemostatic agents, but clinically these differences were rather meaningless. For example, in a pig blood loss of 50, 100 or 200 mg despite a four-fold difference is clinically insignificant.

In clinical practice surgeons often use electrocautery or other similar tools to control liver bleeding rather than using hemostatic agents. This also makes the model rather artificial.

Would this product work in a rapidly bleeding model of lethal hemorrhage?

*We thank the reviewer for the time and expertise to provide helpful comments, and we completely agree with the reviewer's point. As a commonly utilized animal model in literature and industry for evaluating hemostatic efficiency of hemostatic dressings (e.g. Adv. Mater. **2018**, 30, 1700859; Med. Devices **2017**, 10, 273; Int. J. Biol. Macromol. **2015**, 75, 322; Mater. Lett. **2017**, 97, 150), the acute liver punch/abrasion method allowed the comparison of the hemostatic efficiency of the developed dressings against other hemostatic agents studied in this manner. However, we have modified the manuscript to highlight that the model represents a non-lethal and rather modest injury to the liver, and thus cannot be generalized to all forms of bleeding from other sites. Furthermore, we have addressed that future work will include testing the dressings in models of lethal hemorrhage to simulate clinical settings and to explore the clinical significance of the developed dressings (pp. 13 and 21).*

Summary of figure changes

Revised Figure	Previous Figure	Modification
Fig. 3d	Fig. 3d	Corrected data
Fig. 3h	n/a	Added summarized data
Supplementary Figure 1d	n/a	Added additional SEM image
Supplementary Figure 4	Supplementary Figure 4	Increased image brightness

REVIEWERS' COMMENTS:

Reviewer #1 (Remarks to the Author):

The revised manuscript seems to be accepted.

Reviewer #2 (Remarks to the Author):

The authors well addressed my concerns.
